# Potential Drivers for the Re-Emergence of Canine Leptospirosis in the United States and Canada

**DOI:** 10.3390/tropicalmed7110377

**Published:** 2022-11-14

**Authors:** Amanda M. Smith, Jason W. Stull, George E. Moore

**Affiliations:** 1 College of Veterinary Medicine, The Ohio State University, Columbus, OH 43210, USA; 2 Atlantic Veterinary College, University of Prince Edward Island, Charlottetown, PE CIA 4P3, Canada; 3 College of Veterinary Medicine, Purdue University, West Lafayette, IN 47907, USA

**Keywords:** leptospirosis, canine, drivers, triad, re-emerging, vaccination

## Abstract

Canine leptospirosis is an important zoonotic disease in many countries. This review examines potential drivers for increased diagnoses of canine leptospirosis in the United States and Canada, using the epidemiologic triad of agent-environment-host as a template. *Leptospira* spp. are classified into more than 250 serovars, but in many laboratories only 6 are routinely tested for in serologic agglutination tests of canine sera. Leptospiral infections in dogs may potentially go undetected with unemployed or currently employed diagnostic methods. Disease transmission from infected reservoir hosts usually occurs via urine-contaminated environmental sources such as water. Direct contact between infected and susceptible individuals, environmental factors such as climate changes in temperature and/or rainfall, and increasing number and urbanization of reservoir hosts may greatly increase dog exposure risks. A dog’s lifestyle may influence exposure risk to leptospirosis, but vaccination based on proper identification of circulating serogroups dramatically reduces post-exposure infections. Regrettably, resistance to vaccination by veterinarians and dog owners leaves a large number of dogs at risk for this zoonotic disease.

## 1. Introduction

Leptospirosis is a potentially fatal bacterial disease that affects humans and many animal species. The bacteria are widespread in nature across the globe and leptospirosis is considered endemic in most regions. Leptospirosis outbreaks often occur in people from environmental exposure during natural disasters that result in heavy rainfall and flooding [1,2]. Small wild mammals (e.g., rats and mice) are common reservoir hosts, and transmit leptospires into the environment through infective urine. Many wild and domesticated animals however can serve as hosts, but dogs and humans are generally considered incidental hosts. Canine leptospirosis has been long recognized in the United States and Canada [3,4] although the prevalence can vary geographically within countries. Concerns have been expressed in recent years about an increase or re-emergence of canine leptospirosis [5,6,7,8,9]. Due to a lack of historic or current canine leptospirosis prevalence and incidence data, and uncontrolled confounders impacting observational studies, the re-emergence of canine leptospirosis is largely anecdotal. Not only does canine leptospirosis impact canine health, but it can impact human health with the potential of zoonotic transmission in settings such as the home, dog care facilities (e.g., groomers, daycares, boarding facilities), other locations where dogs are permitted to visit (e.g., human healthcare facilities), and veterinary hospitals. As human to human transmission is extremely rare, almost all human cases of leptospirosis occur from direct or indirect contact with animals and their urine [2]. Cases of canine leptospirosis can also serve as a sentinel for increased environmental exposure, indicating increased risk for humans as well [10]. Although exact reasoning behind leptospirosis re-emergence in canines is unknown, there are multiple potential factors for this possible trend. The intention of this narrative review is to explore theorized drivers of the re-emergence of canine leptospirosis in the United States and Canada, using the epidemiologic triad as a template. Further attention to such drivers in the future may prove useful in reducing dog and human risks.

## 2. Epidemiologic Triad

**Figure 1:**
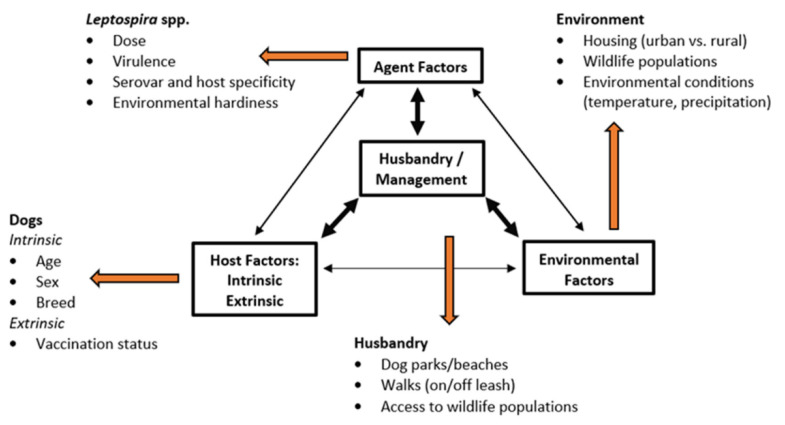
An epidemiologic triad of canine leptospirosis.

One of the basic models of causation of infectious disease is the epidemiologic triad. The epidemiologic triad consists of three main parts–the pathogen or infectious agent, the susceptible host (i.e., dogs in this review), and the environment which bring host and agent together. It is also a model that allows for evaluation of the interactions between the factors that drive the spread of an infectious disease. Figure 1 illustrates an epidemiologic triad for canine leptospirosis (Figure 1).

This triad will serve as the outline for the review of potential disease drivers, and will follow the order more naturally related to *Leptospira* transmission, namely the agent, environment, and host.

## 3. Agent

*Leptospira* spp. are aerobic Gram-negative spirochetes. For many years the two main species were *L. interrogans* (pathogenic strains) and *L. biflexa* (saprophytic strains), until description of an “intermediate” cluster of strains [11]. Nevertheless, the intermediate species exhibit moderate pathogenicity in both humans and animals [12]. Serotyping served as a basis for categorizing species into serovars, and serovars have been grouped into approximately 30 serogroups for epidemiological purposes. There are over 250 recognized serovars in the species *L. interrogans*, and it is likely many remain undiscovered [12,13]. Molecular techniques, including whole-genome sequencing (WGS) and next-generation sequencing (NGS), allowed reclassification of *Leptospira* into different genomospecies; however it is possible for a single genomospecies to include both pathogenic and non-pathogenic serovars [2,14]. The genomospecies are currently separated into 2 clades (pathogenic [P] and saprophytic [S]), each with 2 subclades (P1, P2, S1, and S2) [12,14]. Unlike newer or other taxonomic grouping, serogroups are not defined by DNA similarities, but are groups of antigenically related serovars based on surface antigens. Identification of the infecting serovar is important epidemiologically, but culture of leptospires is often difficult and slow. PCR tests are used in disease diagnosis but fail to identify the causative leptospiral serovar. Serologic testing for antibodies based on serogroup, preferably noted as a four-fold rise in titers, has been a long standing means of identifying infection in animals; but cross-reactions between serogroups makes this method imprecise [14,15]. The number of serogroups tested by a laboratory are often limited due to logistical requirements for conducting the tests, and new serovars in a region may not be properly detected without isolation and molecular identification methods. Advancements in sequence typing will improve serovar and strain identification in the future [14], but current limitations may hinder proper detection of disease in animal species. Serovars also differ in their host species susceptibility, severity of clinical disease, and geographic spread [16]. Immunity to the disease is mainly humoral immunity, and is often serovar specific [13,17].

The most common serovars that are identified as infecting dogs in North America and Europe are Bratislava (maintenance hosts: rat, pig, horse, hedgehog), Canicola (dog), Hardjo (ruminant animals), Icterohaemorrhagiae (rat), Pomona (cattle, pig, skunk, opossum), Grippotyphosa (raccoon, skunk, vole, opossum, cattle), and Autumnalis (mouse) [18]. These serovars have been believed to produce short term urinary shedding in the dog as an incidental host, with the exception of serovar Canicola, for which the dog is the maintenance host and shedding can last for up to two years; but this belief has been challenged with more recent studies documenting possible chronic infections in asymptomatic dogs [18,19,20,21]. Serovars that cause disease in dogs differ in their geographic spread, in part due to the geographic distributions of reservoir hosts. Spatial clusters of serovar Grippotyphosa have been found in Texas and the midwest (Illinois, Michigan, Indiana, Ohio) [22]. A spatial cluster of serovar Pomona has also been found in Texas, and spatial clusters of serovar Icterohaemorrhagiae have been found in the Pacific northwest (California, Oregon) and the midwest (Indiana, Michigan, Ohio) of the US [22]. Nevertheless, spatiotemporal evaluations have documented a geographically diverse range for canine leptospirosis including focal outbreaks in traditionally arid regions [14,23,24,25]. A knowledge of infecting serovars is important for better understanding of disease transmission, reservoir hosts’ roles, and which serovars should be addressed in vaccination programs.

## 4. Environment

Infective leptospires are transmitted in urine from asymptomatic or symptomatic individuals into the environment. Transmission most commonly occurs from contact with leptospires in urine-contaminated water or soil [13,26], thus the environment of natural reservoir hosts is a major contributor to the spread of leptospirosis to incidental hosts such as dogs.

### 4.1. Climate Change

The world’s climate is changing rapidly, which could have devastating effects, including the proliferation of infectious diseases. Climate change includes temperature changes, precipitation changes, and changes in the frequency and severity of natural disasters [27]. Annual temperature in the United States has increased by 0.7 °C between 1986 and 2016, and annual average temperatures are expected to continue rising [27]. Additionally, extreme temperatures are expected to increase, with less extreme cold events and more extreme heat events (e.g., heat waves). The frequency of heavy precipitation events has risen, and the frequency and intensity of these events are expected to increase. Additionally, it is expected that climate change will increase the frequency and severity of extreme storms (e.g., hurricanes, tropical cyclones). Climate change is expected to influence many different diseases due to increased survivability of some pathogens, and changes in host survivability and proximity to human habitats [28,29].

Leptospirosis is an environmentally and seasonally influenced disease and it is expected that climate change will increase the prevalence of this disease in human and animal populations. Canine leptospirosis peaks in late summer and early fall in many areas of the United States and Canada [8,30]. These seasonal trends are thought to be directly related to the bacteria’s optimal survival in high temperatures and stagnant water. Human leptospirosis epidemics have been correlated with high rainfall, and environmental temperature and humidity [1]. A significant positive correlation between number of canine leptospirosis cases and average rainfall three months prior to diagnosis has been found, and average rainfall can predict the occurrence of canine leptospirosis in the United States [30]. Many large-scale human leptospirosis epidemics have occurred after monsoons due to heavy rains and flooding [31]. An increase of large-scale human leptospirosis outbreaks have also occurred due to an increase in natural disasters [32]. Flooding is the most common natural disaster in both developing and developed countries and has been increasing in frequency [33]. A case–control study of human leptospirosis found a 15-fold risk of leptospirosis from walking through flooded waters in Nicaragua following a severe weather event [34]. It is thought there is a similar increase in risk of leptospirosis for canines during severe weather events and natural disasters to that of humans. Frequently flooded areas near dog’s home locations have been identified as a factor increasing risk 4-fold for canine leptospirosis [35].

Large cities are prone to flooding due to large scale impermeable areas (e.g., concrete sidewalks, yards), and many are situated near large rivers or coasts for economic reasons [36]. Additionally, excess debris clogs up drainage systems, contributing to flooding [37]. As mentioned previously, flooding aids in transmission of *Leptospira* spp. and can increase the likelihood a dog will come into contact with the bacteria. In an urban environment, flooding may be more widespread, and it may be difficult to avoid flooded areas.

Urban areas also suffer from the heat island effect. Urban heat islands are when urban areas experience elevated temperatures compared to their rural surroundings [38]. A city can be 1 to 3 °C warmer than surrounding areas annually, and in the evenings this difference can be as great as 12 °C [39,40]. Such heat islands form from the loss of vegetation and increase in paved ground and buildings, which results in less ground cover and reduced evaporation [38]. This urban heat island effect could increase survival of *Leptospira* spp. in an urban environment, much like the effect of increasing temperatures from global climate change.

In terms of disease transmission, climate change directly impacts ecological changes (e.g., biodiversity loss, nutrient cycle changes), sociological changes (e.g., animal and human migration), and changes in transmission biology (e.g., vector and pathogen dynamics) [41]. Climate change can impact reservoir species dynamics directly or indirectly through ecological changes such as habitat loss and changes in food availability. Rodents and peri-domestic wildlife might become displaced during climate events and encroach further into human habitats for food and shelter. Although severe weather events might decimate rodent and wildlife populations, the movement into human habitats for survival will increase their presence in high human traffic areas. This puts them in close contact with domestic dogs and there might be a greater prevalence of wildlife shedding *Leptospira* spp. in or near the dog’s home environment. Increases in rodent or wildlife reservoir populations, or movement of these populations into human habitats due to climate change are likely to increase the likelihood of a dog coming into contact with one of these animals directly or with *Leptospira* spp. bacteria in the environment [29].

In addition to changes in reservoir species dynamics, climate change can also alter pathogen dynamics. *Leptospira* spp. are able to survive for longer periods of time in higher temperatures and humidity [13]. Therefore, increases in average temperatures are predicted to increase pathogen survival in the environment. Additionally, increased rainfall can alter pathogen transmissibility by moving leptospiral organisms from the soil (due to shedding from wildlife and livestock reservoir species) into floodwaters, resulting in greater opportunities for contact with contaminated water [30]. High rainfall and flooding create increased opportunities for canine exposure and transmission (e.g., puddling of water on sidewalks, etc.) [35]. *Leptospira* spp. are also hardy and are able to change their host specificity and virulence in response to environmental pressures [13].

Lastly, climate change can directly affect sociological changes. Higher temperatures are optimal for many canine activities (e.g., swimming, dog park use) that might increase contact with the bacteria. Even if a dog is not participating in canine activities, high rainfall can increase opportunities for contact with contaminated water in their home environment, such as puddling of water in backyards.

### 4.2. Urbanization

Urbanization is formally defined as the process where large numbers of people become permanently concentrated in relatively small areas, forming cities. The United Nations predicts that 68% of the world’s population will live in urban areas by 2050 [42]. In contrast, one hundred years ago only 20% of the world’s population lived in cities [43]. Urbanization has been linked to economic growth and better opportunities and healthcare for those who settle in an urban area [44]. However, the rise of urban areas can also create an ideal setting for emerging and re-emerging infectious diseases such as leptospirosis [45,46,47]. Pathogens that spread from rural to urban settings and adapt to an urban environment can create a great burden as the environmental component of the epidemiological triad becomes altered. The pathogen can now spread in a more rapid manner and evade health care providers unfamiliar with the disease [48]. Canine leptospirosis has traditionally been considered a disease of rural dogs in the US [5,49], but it appears to be increasing in prevalence in urban areas [44]. Human household transmission of leptospirosis has been identified in urban slum communities [50]. Dogs living in urban areas in the United States and Canada have been found to be at increased risk for leptospirosis compared to dogs in suburban or rural areas [24,51,52,53]. Prevalence mapping and spatial analysis of canine leptospirosis cases in the United States have found disease clustering in major US cities such as Chicago, Detroit, and Dallas/Fort Worth [22,54]. However, it is unknown if canine leptospirosis prevalence really is higher in these areas or if variations in case selection for testing skew these findings. If living in an urban area increases a dog’s risk for leptospirosis, then urbanization could be a driving factor for canine leptospirosis. It has been postulated by others that the re-emergence of leptospirosis and change in epidemiology of infecting serovars may be due to urbanization, as it provides greater opportunity for contact between animals and wildlife reservoirs [55]. Although the actual driving factors for the association between canine leptospirosis and urban environments are unknown, there are many plausible explanations.

Urban infrastructure and built environment differ greatly from neighboring suburban and rural areas. They can be characterized by densely populated living areas, lack of abundant green areas and vegetation, and underground public transportation (e.g., subways). Additionally, due to the characteristics of this built environment and high population densities, urban areas often suffer from poor storm water drainage and inadequate waste and sewer management. Urban city environments create ideal habitats for rodents and other wildlife, especially rats. In New York City, encounters between rats and humans are linked to proximity to public recreation areas and subway lines, and the presence of vacant housing [56]. Living in an area with a high garbage volume or in proximity to numerous restaurants also are built environment characteristics that positively influence the number of rats in New York City [57]. Although the prevalence of leptospirosis in the urban rat population in the United States and Canada is unknown, it is thought they are a main reservoir for the disease in urban areas. In a study in Detroit, approximately 60% of brown rats (*Rattus norvegicus*) were found to be shedding *Leptospira* spp. in their urine [58]. The presence of garbage and sewage is associated with higher numbers of rodents and could increase the risk of leptospirosis. Contact with garbage and sewage have been found to be risk factors for human leptospirosis, especially in urban areas [37]. High availability of garbage and sewage in urban environments increases available food sources for rodents and other wildlife species. In one study, higher rat body weight was significantly correlated with higher renal *Leptospira* spp. loads in naturally infected young black rats [59]. Compared to rats in rural areas, rats in urban areas have a higher growth rate and reach sexual maturity faster due to the greater availability of resources [60]. Therefore, rats thriving in urban environments due to abundant food sources might be shedding leptospires in greater quantity than their suburban and rural counterparts.

In addition to rats, other wildlife species also thrive in urban and surrounding peri-urban environments [61], and contact with these species may be an important route of exposure to dogs [62]. While increased human density would presumably deter wildlife habitation, increased food availability and reduced predation promote population increases of some potential *Leptospira*-host species such as raccoons, skunks, coyote, and deer [61,63,64]. Although a large meta-analysis determined a *Leptospira* infection global prevalence of ~15% in the majority of mammalian families [45], large studies in the US have yielded greater seroprevalence rates for leptospirosis in wildlife [65]. Numerous studies of raccoons, skunks, coyote, and/or deer have documented seroprevalence rates of ~10–50% in many areas of the United States [66,67,68,69,70,71,72,73,74,75] and Canada [76,77,78]. Although seroprevalence rates are typically greater than molecular detection or culture rates of leptospires in kidneys, i.e., serology overestimating active infection, serology may potentially underestimate infection in reservoir species [75]. An abundance of reservoir species and/or shedding in urban and peri-urban areas increases the likelihood that pet dogs will be exposed to the bacteria.

It is also important to note that an urban dog likely differs in daily activities and exposures from a rural or suburban dog. High population density and close contact between people in urban areas contribute to the spread of infectious diseases [44]. The same might be said for the dogs that live alongside humans in urban areas. Since urban dwellers often do not have their own backyards, dogs living in urban areas often are exercised, and relieve themselves in common areas such as communal green areas and dog parks. Due to the high abundance of reservoir species in urban areas, there might be high prevalence of leptospires in these public areas, especially when flooded (e.g., many puddles on sidewalks). However, the actual risk to an urban dog due to these lifestyle factors is unknown.

The importance of urbanization as a driver of canine leptospirosis transmission is supported by the finding in multiple studies that living in an urban environment is a risk factor for canine leptospirosis [51,52]. Although aspects contributing to transmission in urban environments have been discussed (abundance of rodent and wildlife species due to livability of the built urban environment, high flooding and temperatures, and unique lifestyles of urban dogs), the primary drivers of transmission in an urban environment are still unknown. Urban canine leptospirosis is an important area to focus future research due to rapid urbanization across the globe, and uncovering transmission dynamics of canine leptospirosis in an urban environment may aid in research of other canine infectious or zoonotic disease research.

### 4.3. Canine Importation, Feral Animals, and Wildlife Trade

Travel and trade might influence the re-emergence of canine leptospirosis in the United States and Canada in two ways–change in biodiversity and introduction of new serovars. Feral animals change the biodiversity in a region and the prevalence of reservoir hosts for leptospirosis, potentially shedding leptospires in the environment. Several states in the US have documented increases in feral swine in the last decade, with seroprevalence indicating past or present leptospiral infections in approximately 50% of populations in some areas [79,80,81,82,83,84,85]. The wildlife trade can also change biodiversity by introducing non-native species and foreign pathogens into the United States [86]. As mentioned previously, a loss of biodiversity can drive pathogen transmission. Due to differing geographic spread of pathogenic *Leptospira* spp. serovars, canine importation raises the possibility of foreign domestic dogs introducing serovars not previously identified, nor currently tested for, in canines in the United States or Canada. It is unlikely wildlife trade and canine importation are main drivers for the spread of canine leptospirosis, but they are still important potential drivers to monitor. Further, these drivers could allow for introduction of a serovar, with one or more of the other drivers increasing prevalence among native wildlife and North American dog populations.

Over the past 15 years, over 3.2 billion live organisms have been knowingly imported into the United States [86]. There were 246,772 mammals representing 190 genera known to be imported into the United States from 2000 through 2005. Of these, 35 genera (18%) were capable of importing *Leptospira* spp. into the United States [87].

Dogs are not among the most common species imported into the United States [87]. However, a large number of dogs are still imported into the United States each year by rescue groups or by owners relocating to the United States. The importation of rescue dogs from different parts of the world has become a trend [88]. The United States Department of Agriculture currently requires all dogs to be vaccinated against leptospirosis when imported for adoption or resale [89]. However, the current vaccine available in the United States only covers four common serovars in the United States–Canicola, Icterohaemorrhagiae, Pomona, and Grippotyphosa [18]. There is thought to be limited or no cross-protection between *Leptospira* spp. serogroups [7]. Due to asymptomatic shedding, limitations of the required vaccine, and the presence of varying serovars in other parts of the world, it is possible that dogs imported into the United States could drive canine leptospirosis through the introduction of a new serovar to the United States. A study of >400 canine serum samples from dogs in Ireland found serogroup Ballum was the most common serogroup to which dogs had antibodies [90]. Serogroup Ballum has previously been identified in the United States, but it is not thought to be a common serovar impacting canines in the United States [91]. Dogs on the Caribbean island of Saint Kitts had highest seroprevalence for common United States serovars Autumnalis and Icterohaemorrhagiae, but they also had seroprevalence for serovar Djasiman which is not common in the United States [92]. The most common serogroup in a study of >200 canines in Thailand was Sejroe, which is also not considered a common canine serogroup in the United States [93]. Although leptospirosis infection may be suspected based on clinical signs before a dog is imported into the United States, dogs have been found to be asymptomatic leptospiral carriers, or exhibit vague clinical signs which may be overlooked, and thus could import serovars not covered by the locally available vaccine [94,95]. Additionally, although documented vaccination is a requirement for some imported dogs, it is unknown if imported dogs are sufficiently vaccinated. A study comparing rabies antibody titers of dogs vaccinated in Finland and street dogs imported from the Russian Federation and Romania to Finland and vaccinated in their home countries found the imported dogs did not have sufficient rabies antibody levels [96]. Possible explanations included improper use or storage of the vaccine, or falsified documents and no actual history of vaccination [96]. These factors could also be a concern for canine leptospirosis vaccinations performed abroad.

Additional research is needed to establish the current prevalence of canine leptospirosis serovars circulating in the United States and to test imported dogs with a broad serovar panel to capture potential importation of pathogenic canine serovars. Although the six common serovars infecting canines in the United States are widely assumed, due to the poor serovar predictability of the MAT, the pathogenic serovars currently infecting the canine population in the United States and Canada are largely unknown. A commercial serovar-specific test with high sensitivity and specificity is needed.

## 5. Host

### 5.1. The Evolving Role and Lifestyle of the Dog

Over time, dogs have continued to become increasingly integrated into human life. Approximately 40% of US households and 35% of Canadian households own dogs and approximately 67% of dog owners in these countries consider their dogs to be family members [97,98]. Some people view dogs as humanistic, or surrogate humans [99,100]. Dogs have also become surrogate children for some–with higher attachment to a dog associated with being a female owner and not having children [101]. Due to these evolving views and individuals’ tendency to humanize their dog, people are going to great lengths to meet the perceived needs of their dogs. Dog parks, dog beaches, boarding kennels labelled as pet resorts, and dog daycares are common canine group settings in the United States, which result in high dog-dog direct and indirect interactions, involving dogs from a wide geographic area. Such settings likely result in additional exposure opportunities to numerous pathogens, including leptospirosis. A recent canine leptospirosis outbreak in Arizona, which is not known to be a high-risk location for canine leptospirosis, stemmed from dog daycare and boarding facilities [25,102]. These settings could continue to act as likely locations for canine infectious disease outbreaks. In addition to potentially being at higher risk for canine infectious diseases, dogs frequenting these locations might likely spread disease in their community. Akin to a human contracting a pathogen on a densely packed airplane and introducing it to their community, dogs frequenting multiple canine group settings over a short period of time (e.g., daycare during the week, dog parks on Saturday, dog beaches on Sunday, conformation or agility shows) could potentially infect many more dogs compared to an average dog. In the case of leptospirosis, this can be true during an outbreak when a dog might be asymptomatically shedding the bacteria into multiple environments.

The positive role of a dog as a companion has been widely accepted, with various groups of individuals benefiting from this human–animal bond. One such group is individuals who are experiencing poverty and homelessness. Research has found that the bond between an individual experiencing homelessness and their companion animals is strong, and sometimes stronger than that of the general population [103,104]. Nonprofit groups have worked to preserve this bond, offering supplies and veterinary care so these dogs can stay with their owners. Regardless, dogs owned by individuals experiencing poverty and homelessness raises an interesting and important consideration when thinking about canine leptospirosis prevalence. Those experiencing poverty in developed urban centers often suffer from poor standards of living, and homelessness. Impoverished urban populations often have inadequate housing, and poor sanitation, which can promote rat infestations and facilitate pathogen transmission through close contact with rodents [105]. Numerous zoonotic infections have been identified among urban homeless and marginalized populations in the United States, including leptospirosis [106]. In the 1990s, 16% of people from the inner city of Baltimore, MD were seropositive to *Leptospira* spp. and seropositivity was associated with low income [107]. It is reasonable to assume that the increased risk of exposure to leptospirosis for impoverished and homeless populations is similar for the dogs they own. Preserving the human–animal bond for these populations is important, but the risk of canine leptospirosis should be considered when planning, and prevented when possible. However, dog ownership statistics for individuals experiencing poverty and homelessness in the United States and Canada are limited [108]. Research is needed to investigate this unique population as these individuals are at higher risk for infectious and zoonotic diseases, and their dogs may have increased exposure risk too.

### 5.2. Role of Vaccination

Dogs are considered incidental hosts for leptospirosis in the US and Canada although, as previously noted, they can be reservoir hosts for serovar Canicola [25,109,110]. Canine leptospirosis vaccination is an effective prevention measure based on experimental challenge and observational assessments [111,112,113]. Although immunity reportedly lasts for 12 months in experimental studies, and serovar specific immunity has been found to last up to fifteen months (serovar Grippotyphosa) experimentally, this protection is not absolute as leptospires have been isolated from vaccinated dogs [21,114,115,116]. It is unknown specifically how long immunity lasts when challenged with natural leptospirosis infection by different vaccine serovars. Generally, there is not full cross-protection against other serovars, but some studies have shown partial immunity to other serogroups [117]. Leptospirosis vaccines are killed bacterins, requiring two initial doses to produce protective immunity and annual re-vaccination thereafter. Although the vaccines are highly effective, vaccine failures may occur [118].

Although leptospirosis vaccines were typically administered to all dogs several decades ago as part of a canine distemper-hepatitis-leptospirosis (DHL) combination, recent professional vaccination guidelines in the United States have not recommended their use on all dogs, i.e., it is considered “non-core” [119,120]. The prevalence of leptospirosis vaccination in dogs in the United States and Canada is unknown but a recent study of >1500 US veterinary hospitals found a range of 0–100% (median: 70%) vaccination rate for leptopsirosis by hospitals in dogs also receiving core vaccines, e.g., rabies vaccine [121]. Failure to vaccinate dogs leaves them susceptible to infection. Most cases in a case–control study in California were not vaccinated against canine leptospirosis [62], and in outbreaks in Arizona in the last decade dog owners reported they did not know there was vaccine against leptospirosis or their veterinarians did not recommend it [25]. A potential explanation for low vaccination is vaccine hesitancy and concern about adverse vaccine events. A large observational vaccine field study (n = 130,557 dogs) found the risk of adverse events was higher when a dog received a leptospirosis vaccine, but the occurrence of these adverse events was still low (incidence rate of owner-reported adverse events in dogs administered a leptospirosis vaccine was 53.0/10,000, or 0.53%) [122]. However, there was not a significant difference between the leptospirosis vaccine and other vaccines when it came to severe and life-threatening adverse events, and another study found that vaccines with leptospirosis included were not more reactive than vaccines without the leptospirosis component [123]. A lack of vaccination or ineffectiveness of a vaccine could drive infection due to a large susceptible canine population.

Conversely, effective vaccination might also drive leptospirosis by placing selective pressure on the circulating serovars. For example, serovar Autumnalis has emerged and has been newly implicated in clinical canine disease [124]. This serovar is not covered in the current 4-way vaccine available in the United States and Canada, and it is theorized that effective vaccination against the serovars included in the vaccine have allowed for emergence of serovar Autumnalis [18]. Testing with improved ability to accurately detect infecting serovars is needed to understand what serovars are circulating in the canine population to understand the effects of vaccination.

### 5.3. Actions by Dog Owners

This review has explored a number of potential drivers of canine leptospirosis, however for dogs living specific lifestyles and participating in certain activities the variation in exposure risk is still largely unknown. However, several preventative activities by pet owners are theorized to lower a dog’s risk of contracting leptospirosis, and thus increased public knowledge of appropriate preventive medicine measures may impact the likelihood of clinical disease in dogs. Owner education on public health disease risks, particularly waterborne diseases, may decrease the incidence of leptospirosis. As climate change increases the number of severe weather events and the world experiences greater precipitation and higher temperatures, dog owners may or may not be aware of disease risks associated with stagnant water [125].

Failure to control rodent populations can lead to numerous public health disease threats [126]. Dog owner education, with appropriate actions, are necessary to reduce the presence of rodents in and around a home whether in urban, peri-urban, or rural areas. One aspect of the dog’s environment seldom considered, even by veterinarians, is the presence of backyard poultry which often attract rodent populations to chicken feed and litter [127].

Knowledge of veterinary guidelines by dog owners and business owners can help impact infectious disease transmission in both individual and canine group settings [128]. If a dog has clinical evidence of an infectious disease, the owner should seek veterinary care and not let the dog have contact with dogs outside of the household or partake in group activities. Dog business owners should consider canine vaccination requirements, routinely clean and disinfect their facilities, and prevent the entry of rodents or wildlife into the facility or outdoor areas the dogs frequent [128].

Lastly, the most effective measure by owners to prevent canine leptospirosis is leptospirosis vaccination. Although it may not cover all potential infective serovars, vaccination will significantly lower a dog’s risk for infection and shedding for many of the common serovars that infect canines in the United States and Canada. Regrettably, recent public sentiment concerning the safety of vaccination in general has impacted these same geographical areas, producing vaccine-resistant or -concerned clients [129,130]. Thus dog owners may not take veterinarians’ recommendations, or veterinarians may be hesitant to encourage vaccination to prevent disease and its possible transmission.

## 6. Conclusions

Leptospirosis like other infectious diseases is greatly influenced by the epidemiologic triad of agent, environment and host. These factors and their interactions are dynamic, affecting both increases and decreases in disease incidence in different populations. A knowledge of infecting serovars is important for better understanding of the agent’s transmission, reservoir hosts’ roles, and which serovars should be addressed in vaccination programs. Environmental factors may currently promote an increase is disease exposure risk to dogs in the United States and Canada, while resistance to canine vaccination may also allow an increase in clinical cases of this zoonotic disease.

## Data Availability

Not applicable.
